# Auditory grouping is necessary to understand interrupted mosaic speech stimuli

**DOI:** 10.1121/10.0013425

**Published:** 2022-08-15

**Authors:** Kazuo Ueda, Hiroshige Takeichi, Kohei Wakamiya

**Affiliations:** 1 Department of Human Science, Faculty of Design/Research Center for Applied Perceptual Science/Research and Development Center for Five-Sense Devices, Kyushu University, 4-9-1 Shiobaru, Minami-ku, Fukuoka 815-8540, Japan; 2 Open Systems Information Science Team, Advanced Data Science Project (ADSP), RIKEN Information Research and Development and Strategy Headquarters (R-IH), RIKEN, 1-7-22 Suehiro-cho, Tsurumi-ku, Yokohama, Kanagawa 230-0045, Japan; 3 Department of Communication Design Science, Faculty of Design, Kyushu University, 4-9-1 Shiobaru, Minami-ku, Fukuoka 815-8540, Japan

## Abstract

The intelligibility of interrupted speech stimuli has been known to be almost perfect when segment duration is shorter than 80 ms, which means that the interrupted segments are perceptually organized into a coherent stream under this condition. However, why listeners can successfully group the interrupted segments into a coherent stream has been largely unknown. Here, we show that the intelligibility for mosaic speech in which original speech was segmented in frequency and time and noise-vocoded with the average power in each unit was largely reduced by periodical interruption. At the same time, the intelligibility could be recovered by promoting auditory grouping of the interrupted segments by stretching the segments up to 40 ms and reducing the gaps, provided that the number of frequency bands was enough ( ≥4) and the original segment duration was equal to or less than 40 ms. The interruption was devastating for mosaic speech stimuli, very likely because the deprivation of periodicity and temporal fine structure with mosaicking prevented successful auditory grouping for the interrupted segments.

## I. INTRODUCTION

It has been well-known that speech signals are highly redundant; therefore, speech perception is robust against severe degradations in the temporal and frequency domains. Periodic interruption (e.g., Kidd and Humes, 2012; Miller and Licklider, 1950; Powers and Speaks, 1973; Powers and Wilcox, 1977; Shafiro *et al.*, 2018; Shafiro *et al.*, 2016; Ueda and Ciocca, 2021; Ueda *et al.*, 2021) and local time reversal (e.g., Greenberg and Arai, 2004; Matsuo *et al.*, 2020; Saberi and Perrott, 1999; Steffen and Werani, 1994; Stilp *et al.*, 2010; Ueda and Ciocca, 2021; Ueda *et al.*, 2017; Ueda *et al.*, 2019; Ueda and Matsuo, 2021) are among the degrading techniques that have been performed in the time domain to investigate the influence of degradation on speech perception. Filtering in the frequency domain (e.g., French and Steinberg, 1947; Humes and Kidd, 2016; Miller and Nicely, 1955; Studebaker *et al.*, 1987; Warren *et al.*, 2005) and smearing spectrotemporal modulations (e.g., Drullman *et al.*, 1994a,b; Flinker *et al.*, 2019; Kidd *et al.*, 2009; Nakajima *et al.*, 2017; Shannon *et al.*, 1995; Smith *et al.*, 2002; ter Keurs *et al.*, 1992, 1993; Venezia *et al.*, 2016; Zeng *et al.*, 2004) have also been employed. Here, we report that the intelligibility for spectrotemporally degraded speech stimuli was severely deteriorated with interruption and the intelligibility was recovered when we modified the stimuli in a way to promote auditory grouping.

Miller and Licklider (1950) provided systematic data on the perception of interrupted speech stimuli with a list of phonemically balanced monosyllabic speech stimuli. The study showed that when the percentage of the time that the speech was left on was 50% and the segment duration (for on and off) was gradually extended, the speech intelligibility was around 80% until the segment duration became about 80 ms and the intelligibility declined to about 50% at the 500-ms segment duration. Miller and Licklider (1950) also referred to auditory continuity (Bregman, 1990; Warren, 2008) for the interrupted speech stimuli. They found the *picket fence effect*, i.e., the interrupted speech stimuli sounded as if it were continuous when the silent gaps were replaced with strong noise. Since then, the investigations on the perception of interrupted speech stimuli have been expanded with meaningful speech sentences and filling noise (Cherry and Wiley, 1967; Holloway, 1970; Powers and Wilcox, 1977; Wiley, 1968), filtered speech signals and noise (Bashford *et al.*, 1992), listeners with hearing loss (Kidd and Humes, 2012; Shafiro *et al.*, 2016), time-compressed or extended speech stimuli by altering the silent gaps (Shafiro *et al.*, 2016), lowpass-filtered and interrupted auditory stimuli and interrupted visual stimuli (Shafiro *et al.*, 2018), interrupted locally time-reversed speech stimuli and filling noise (Ueda and Ciocca, 2021), and interrupted mosaic speech stimuli (Ueda *et al.*, 2021) to name a few.

Frequency resolution and temporal fine structure can be degraded when a speech signal is noise-vocoded (Shannon *et al.*, 1995): the original signal is bandpass filtered, the amplitude envelopes for the filter outputs are extracted, bandpassed noises of corresponding bandwidths are modulated with the amplitude envelopes, and the modulated bandpassed noises are summed up across frequency. Even though periodicity and temporal fine structure (Rosen, 1992) are removed in noise-vocoded speech, it is still intelligible, provided that the number of frequency bands is enough, i.e., typically four or more (e.g., Dorman *et al.*, 1997; Ellermeier *et al.*, 2015; Kishida *et al.*, 2016; Shannon *et al.*, 1995; Smith *et al.*, 2002; Ueda *et al.*, 2018). Thus, speech can be perceived through only amplitude envelopes in several frequency bands without periodicity and temporal fine structure, which normally provide a grouping cue (Apoux and Healy, 2013; Apoux *et al.*, 2013). On the other hand, a study employing a more flexible vocoding technique, i.e., TANDEM-STRAIGHT (Kawahara and Morise, 2011), provided a piece of evidence showing that periodicity did contribute in improving intelligibility of vocoded speech stimuli with the six-band spectral resolution, when the stimuli were interrupted with the 227-ms segment duration (Clarke *et al.*, 2016). Under rougher or finer spectral resolution conditions, adding periodicity did not improve intelligibility significantly. Furthermore, flattening or exaggerating *F*_0_ contours had no effect on the intelligibility of vocoded-and-interrupted speech stimuli (Clarke *et al.*, 2017).

The methods for determining the frequency division in noise-vocoded speech are important for intelligibility. The typical approach is to use logarithmically equal steps (e.g., Clarke *et al.*, 2016; Greenwood, 1990). Another approach was developed by Plomp and his colleagues (Plomp, 1976, 2002; Plomp *et al.*, 1967; Pols *et al.*, 1973). They used principal component analysis to describe speech spectrum and found that Dutch steady vowels can be represented by only two acoustic principal components (cf. Zahorian and Rothenberg, 1981). Extending this approach, Ueda and Nakajima (2017) used a critical-band filter bank to simulate the frequency analysis function in the auditory periphery and filter 58–200 complete sentences in eight different languages/dialects, i.e., American English, British English, Cantonese, French, German, Japanese, Mandarin, and Spanish, spoken by 10–20 speakers in each language/dialect. A correlation coefficient matrix between power fluctuations in the filtered outputs was submitted to principal component analysis, and the first three (and four) principal components were varimax rotated to yield “factors” of speech (cf. Grange and Culling, 2018; Kishida *et al.*, 2016). The factors for these languages/dialects were very similar to each other. Crossover points on factor loading curves for all languages/dialects examined that were plotted against the filter channels yielded four frequency bands, common to eight languages/dialects. The factor scores of the three factors provided a reasonable curve on which the English phonemic categories were ordered systematically (Nakajima *et al.*, 2017; Zhang *et al.*, 2020). One of the factors highly correlated with *sonority* scales (Nakajima *et al.*, 2017) and the frequency band which corresponded to the factor was critical in maintaining high intelligibility (Matsuo *et al.*, 2020; Ueda and Matsuo, 2021). The four frequency bands produced highly intelligible noise-vocoded speech in German and Japanese (Ellermeier *et al.*, 2015; Ueda *et al.*, 2018). The four frequency bands were also used in investigating frequency specificity in speech perception (Ueda *et al.*, 2018) and perception of checkerboard speech stimuli, in which original speech signals were segmented in frequency and time, and interrupted for every other unit across the two dimensions (Ueda *et al.*, 2021).

**FIG. 1.**
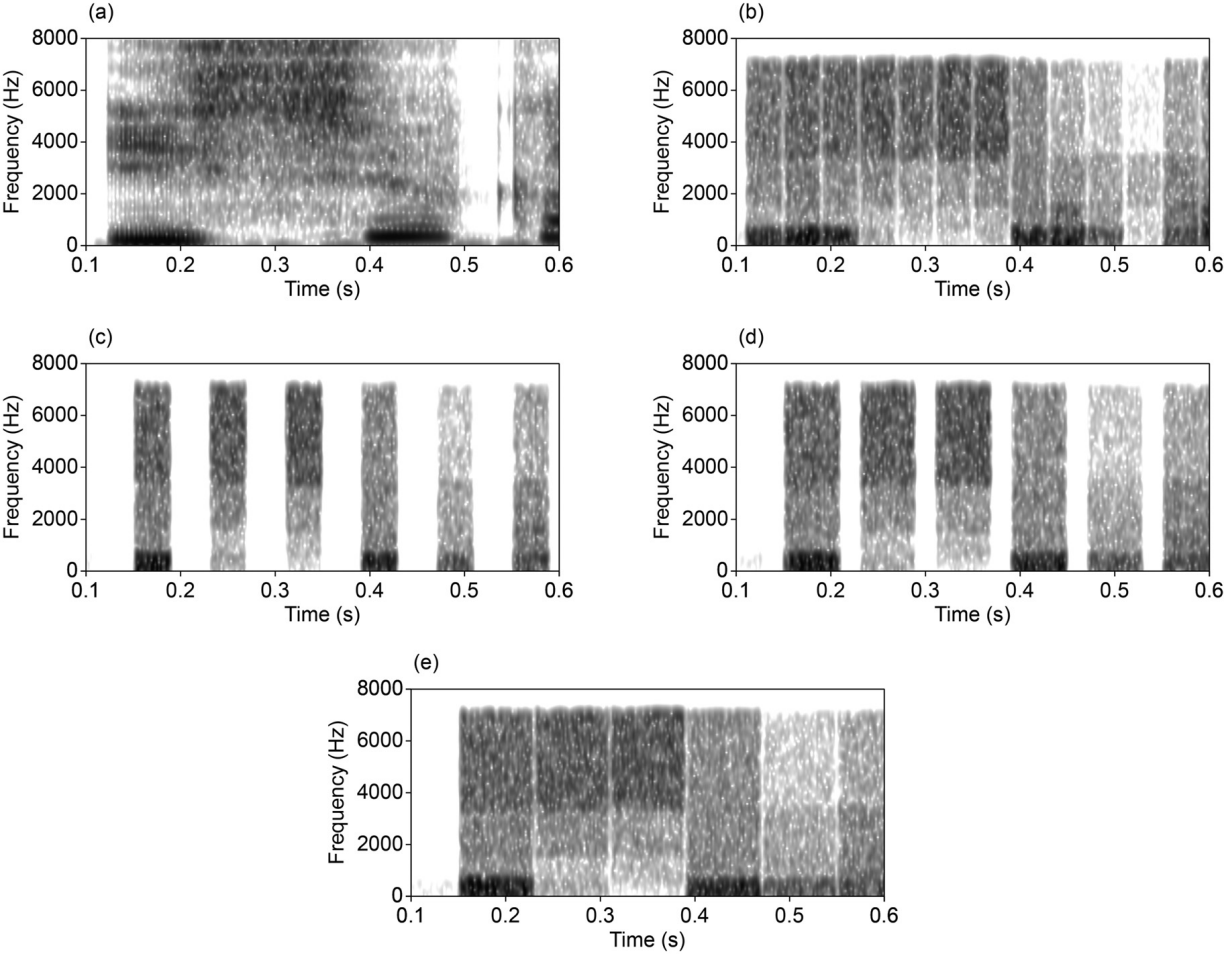
Examples of broadband spectrograms for the stimuli, produced from the same fragment of an original spoken Japanese sentence by a female talker from NTT-AT, “Phonemically Balanced 1000-Sentence Speech Database,” depicting (a) the original speech sample, (b) mosaic speech with four frequency bands (passbands of 50–570, 570–1600, 1600–3400, and 3400–7000 Hz) and the 40-ms segment duration, including 5-ms root-of-raised-cosine ramps in amplitude (four band-noises were modulated with stepwise functions reflecting the averaged power in each frequency-time unit), (c) interrupted mosaic speech, i.e., every other segment was replaced with a silent gap, (d) interrupted and stretched mosaic speech in which each segment was stretched in time by a factor of 1.5 and the silent gaps were shrunk by a factor of 0.5, and (e) interrupted and stretched mosaic speech in which each segment was stretched in time by a factor of 2.0 and the silent gaps were removed.

*Mosaic speech* [Eguchi *et al.*, 2022; Nakajima *et al.*, 2018; Schlittenlacher *et al.*, 2019; Fig. 1(b)] is similar to noise-vocoded speech in the sense that an original speech signal is divided into several frequency bands and a voice source is replaced with band-noises. However, with regard to mosaic speech, the original speech signal is divided not only in frequency but also in time, and the average power within each unit is reflected to a stepwise amplitude envelope in each frequency band, which modulates a band-noise source. The modulated band-noises are summed across frequency to produce mosaic speech.

The first formal publication on mosaic speech (Nakajima *et al.*, 2018) revealed that the intelligibility of the mosaic speech stimuli with 20 frequency bands (critical bands) was more than 95% for the stimuli with the 20- or 40-ms segment duration, whereas it started to decline sharply when the segment duration exceeded 40 ms, went down to about 45% at the 80-ms segment duration, and the intelligibility reached the floor with the segment duration of 320 ms. This general tendency has been confirmed by a follow-up experiment in which Mandarin Chinese and Japanese mosaic speech stimuli were used and speech rates were normalized (Eguchi *et al.*, 2022). Furthermore, another investigation in which the segment duration of mosaic speech stimuli was shrunk, preserved, or stretched strongly suggested that the auditory system needed averaged spectral information within the window of 40 ms for speech perception to attain high intelligibility (Santi *et al.*, 2020).

It is possible to interrupt mosaic speech stimuli as well. The simplest way to interrupt mosaic speech stimuli is to replace every other segment in time with a silent interval of the same duration [Fig. 1(c)]. Thus, we will consider only this way of interruption hereafter. This manipulation preserves half of the original mosaic units and discards the others. Our preliminary data (*n *=* *2) showed that the interruption introduced in mosaic speech stimuli resulted in a devastating effect: the intelligibility decreased to less than 10% except for the stimuli with the 20 frequency bands and the 20-ms segment duration, which brought the intelligibility to 85% (Ueda *et al.*, 2021, experiment 1).

These results give rise to two equally plausible hypotheses at this stage. First, because mosaicking degrades speech cues, depending on the frequency and time resolution, it is possible that although the mosaic speech stimuli with the 20 frequency bands and 40-ms segment duration contains enough speech cues to attain nearly 100% intelligibility, removing half of the segments and replacing with silent gaps might be enough to reduce the available speech cues below the threshold of speech perception, leading to the drastically reduced intelligibility. This hypothesis is referred to as *insufficient speech fragments* hypothesis in this article. Second, because mosaicking removes periodicity and temporal fine structure, it is possible that a poor grouping cue makes grouping the separated segments into a coherent stream difficult. As a result, it becomes difficult to compare spectral differences between the segments and to extract speech cues. This hypothesis shall be called *failed auditory grouping* hypothesis.

At the same time, the possibility of modifying the *mosaicked* segment duration has never been explored in an experiment with interrupted mosaic speech stimuli. If the segment duration of the mosaicked speech segments is stretched and the duration of the silent gaps is shrunk by the same amount, it is possible to produce a variety of stimuli with different durations of stretched segments and silent gaps, keeping the total duration and proportion of the segments derived from original speech unchanged [Figs. 1(c)–1(e)]. There are three possibilities in defining temporal resolution for this kind of interrupted and stretched mosaic speech stimuli: (1) by the original segment duration, (2) by the stretched segment duration, and (3) by the onset-to-onset interval between adjacent segments. If we define temporal resolution by the original segment duration or the onset-to-onset interval between adjacent segments, the temporal resolution is unaffected by stretching. If we define temporal resolution by the stretched segment duration, the temporal resolution is degraded by the manipulation. Under any of these definitions, the stretching never improves the temporal resolution. Thus, if any improvement in intelligibility is observed for the interrupted and stretched mosaic speech stimuli, the improvement should be attributed to any other reason. On the other hand, reducing the silent gap duration should facilitate auditory grouping based on the principle of proximity (Bregman, 1990). However, if the insufficient speech fragments hypothesis is correct, any of these manipulations should not affect intelligibility. By contrast, if the failed auditory grouping hypothesis is correct, stretching the mosaicked segment duration and reducing the silent gap duration should increase intelligibility. In addition, if intelligibility is reduced by stretching, it means that the temporal resolution, which is defined by the stretched segment duration, determines the intelligibility of interrupted mosaic speech stimuli independently of the gap duration reduction or auditory grouping (the *reduced temporal resolution* hypothesis). Thus, by using the technique of stretching mosaicked segments, we can conduct an experiment to determine which of the three hypotheses, i.e., the insufficient speech fragments hypothesis, failed auditory grouping hypothesis, or reduced temporal resolution hypothesis, is the most plausible one to explain the results.

Therefore, the purpose of the current investigation was twofold: (1) to confirm the preliminary results (Ueda *et al.*, 2021, experiment 1), and (2) to test the three hypotheses by examining the effect of stretching the mosaicked segment duration on intelligibility, keeping the total duration unchanged by reducing the silent gap duration.

## II. METHOD

### A. Listeners

A total of 12 paid listeners (20–23 years old) participated in the experiment. They were all Japanese native listeners with normal hearing. Their hearing levels were tested with an audiometer (RION AA-56, RION, Kokubunji, Japan) within the frequency range of 250-8000 Hz. The research was conducted with prior approval of the Ethics Committee of Kyushu University (approval ID, 70). All of the participants gave informed consent in compliance with the protocol approved by the committee. All of the methods employed were in accordance with the guidelines provided by the Japanese Psychological Association.

### B. Conditions

Five steps of the number of frequency bands (2, 4, 8, 16, and 20), three steps of original segment duration (20, 40, and 80 ms), and three steps of stretching ratios (1.0, 1.5, and 2.0) were combined to construct experimental conditions. The passbands for each filter bank are indicated in Table I. The 20-band filter bank was based on critical bandwidths (Zwicker and Terhardt, 1980) in the range of 50–7000 Hz. The four-band filter bank was based on the findings by Ueda and Nakajima (2017) and rounded on the critical bandwidth scale. The 2-, 8-, and 16-band filter banks were based on the four-band filter bank. Each band in the four bands was halved on the critical bandwidth scale to yield the eight bands, which were further halved to the 16 bands. The lower two bands and upper two bands in the four-band setting were combined to produce the two-band setting. In addition, the original (unprocessed) speech stimuli were employed as control stimuli.

**TABLE I.** Passbands for the bandpass filtering in preparing the stimuli. The passbands are expressed in Hz. The 20-band setting was based on critical bandwidths (Zwicker and Terhardt, 1980). The four-band setting was based on the frequency boundaries found by Ueda and Nakajima (2017).

Band No.	20 bands	16 bands	8 bands	4 bands	2 bands
1	50–150	50–175	50–300	50–570	50–1600
2	150–250	175–300	300–570	570–1600	1600–7000
3	250–350	300–425	570–1000	1600–3400	
4	350–450	425–570	1000–1600	3400–7000	
5	450–570	570–770	1600–2320		
6	570–700	770–1000	2320–3400		
7	700–840	1000–1270	3400–4800		
8	840–1000	1270–1600	4800–7000		
9	1000–1170	1600–1925			
10	1170–1370	1925–2320			
11	1370–1600	2320–2800			
12	1600–1850	2800–3400			
13	1850–2150	3400–4000			
14	2150–2500	4000–4800			
15	2500–2900	4800–5800			
16	2900–3400	5800–7000			
17	3400–4000				
18	4000–4800				
19	4800–5800				
20	5800–7000				

Thus, 46 conditions were prepared in total. These conditions were randomly ordered to form a trial block, and five blocks of trials were run for each listener. Consequently, each listener participated in 230 trials in total. A total of 230 different sentences were allotted to the trials for each listener. The combinations of the conditions and sentences were shifted among individual listeners. However, the counterbalancing was imperfect due to the small number of participants compared to the total number of conditions.

### C. Stimuli

A total of 230 Japanese sentences spoken by a professional male talker were extracted from the “Phonemically Balanced 1000-Sentence Speech Database” (NTT Advanced Technology Corp., Kawasaki, Japan; 44 100-Hz sampling, 16-bit linear quantization). The average duration of a sentence was 4.3 s [standard deviation (SD) = 0.93]. The average number of morae (syllable-like units in Japanese) per sentence was 27.5 ( SD=5.45). The speech rate which was calculated with the number of morae per sentence divided by the duration was 6.4 on average. This value was very close to the average in another speech database (NTT-AT, 2002), 6.8, which was calculated over ten speakers (five males and five females).

The original speech samples were passed through one of the bandpass-filter banks (Table I). Each filter was constructed as a concatenate convolution of an upward frequency glide and its temporal reversal [a finite impulse response (FIR) filter]. The frequency characteristics of the filters showed transition regions of 100-Hz wide with out-of-band attenuations of 50–60 dB.

Each filter output was segmented into 20-, 40-, or 80-ms segments, and the average power in each segment was calculated. The series of average values was transformed into a stepwise amplitude envelope with the same segment duration, including 5-ms root-of-raised-cosine ramps. The amplitude envelope in each frequency band modulated corresponding bandpassed noise. The modulated noises were summed up across frequency to yield mosaic speech. Every other segment of the mosaic speech was replaced with a silent gap of the same segment duration, generating an interrupted mosaic speech stimulus of the stretching ratio of 1.0. Then, the mosaicked segment duration was stretched according to the stretching ratio, whereas the silent gap duration was adjusted according to the ratio calculated by [ 2−(stretching ratio)], so that the total duration of the stimulus would be unchanged from the original mosaic speech [Figs. 1(b)–1(e)].

### D. Procedure

The stimuli were presented to participants diotically through headphones (Beyerdynamic DT 990 PRO, Beyerdynamic GmbH, Heilbronn, Germany) in a double-walled sound-attenuated booth (Music cabin SD3, Takahashi Kensetsu, Kawasaki, Japan). Custom software written with the LiveCode package (LiveCode, 2018) was used to present the stimuli. The headphones were driven with a universal serial bus (USB) interface (Roland Rubix24, Roland Corp., Shizuoka, Japan) and amplifier (Luxman L-505f, Luxman Corp., Yokohama, Japan). The sound pressure level of the original speech stimuli was adjusted to 60 dB (A) using a 1000-Hz calibration tone, which was matched with the stimuli in the root mean square value in amplitude. The sound pressure level was measured with an artificial ear (Brüel and Kjær type 4153, Brüel and Kjær Sound and Vibration Measurement A/S, Nærum, Denmark), a condenser microphone (Brüel and Kjær type 4192), and a sound level meter (Brüel and Kjær type 2260).

Participants were instructed to write down exactly what they heard with hiragana or katakana (sets of symbols that are used to represent Japanese morae). They were instructed to write down the morae that they could immediately recognize and not fill blanks afterward from the context. Each mora was examined for whether it was correct or incorrect. The number of correct morae in each sentence was counted. A blank response was counted as incorrect, and homophone errors were permitted. Although statistical analysis was based on the binomial results, i.e., correct or incorrect, the results were represented with percentages of correct morae.

## III. RESULTS

**FIG. 2.**
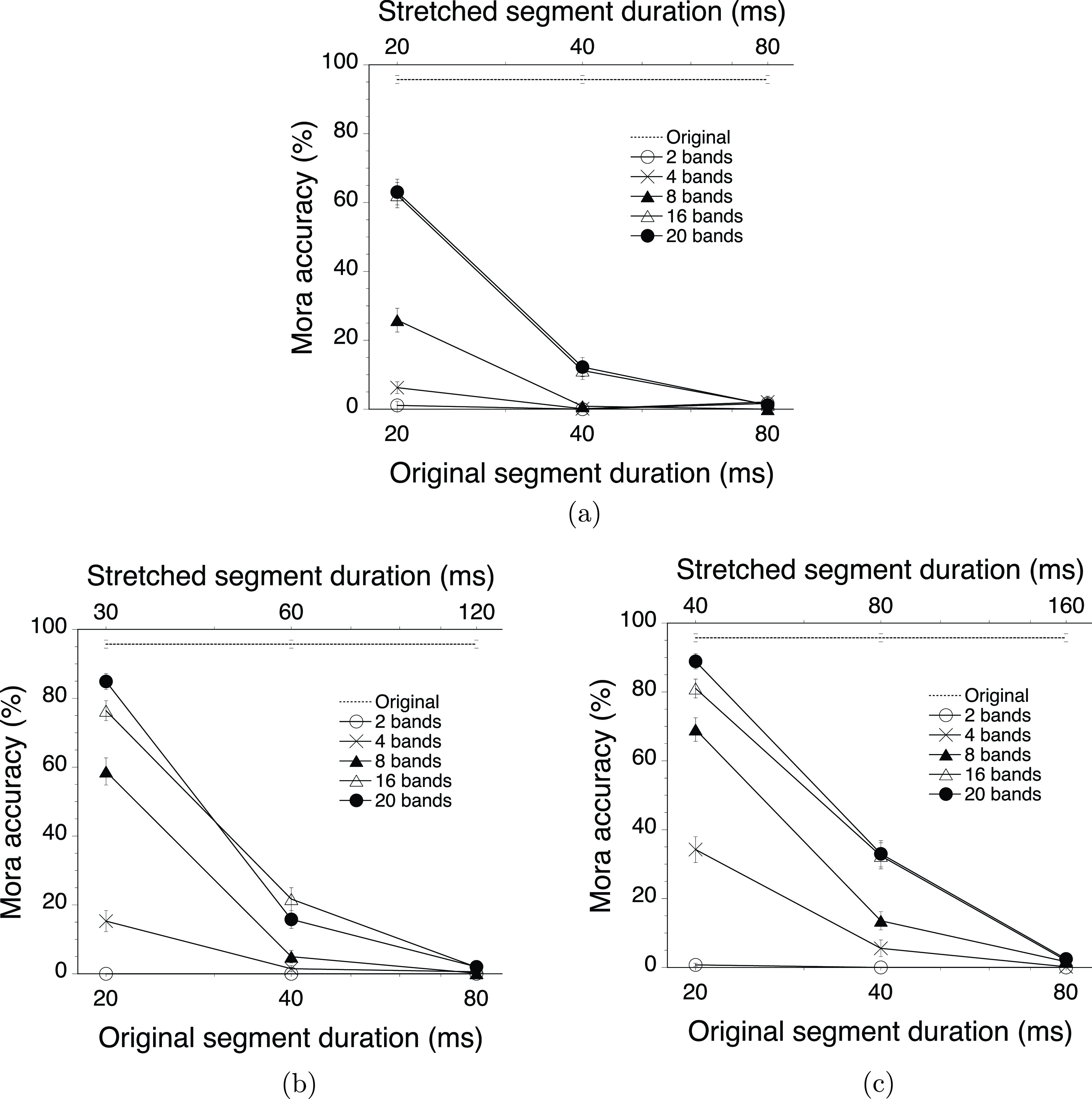
The mora accuracy in percentages as a function of stretching ratio, original segment duration, and number of frequency bands. Panels represent the data for the stretching ratios: (a) 1.0, i.e., no stretching, every other segment was replaced with a silent gap of the same duration; (b) 1.5, e.g., each original 20-ms segment was stretched to 30 ms, whereas each silent gap was shortened to 10 ms; and (c) 2.0, e.g., each original 20-ms segment was stretched to 40 ms and connected to the next segment without any gap. The data for the original stimuli are presented in all panels as a reference. The error bars show the standard error of the mean (SEM).

**FIG. 3.**
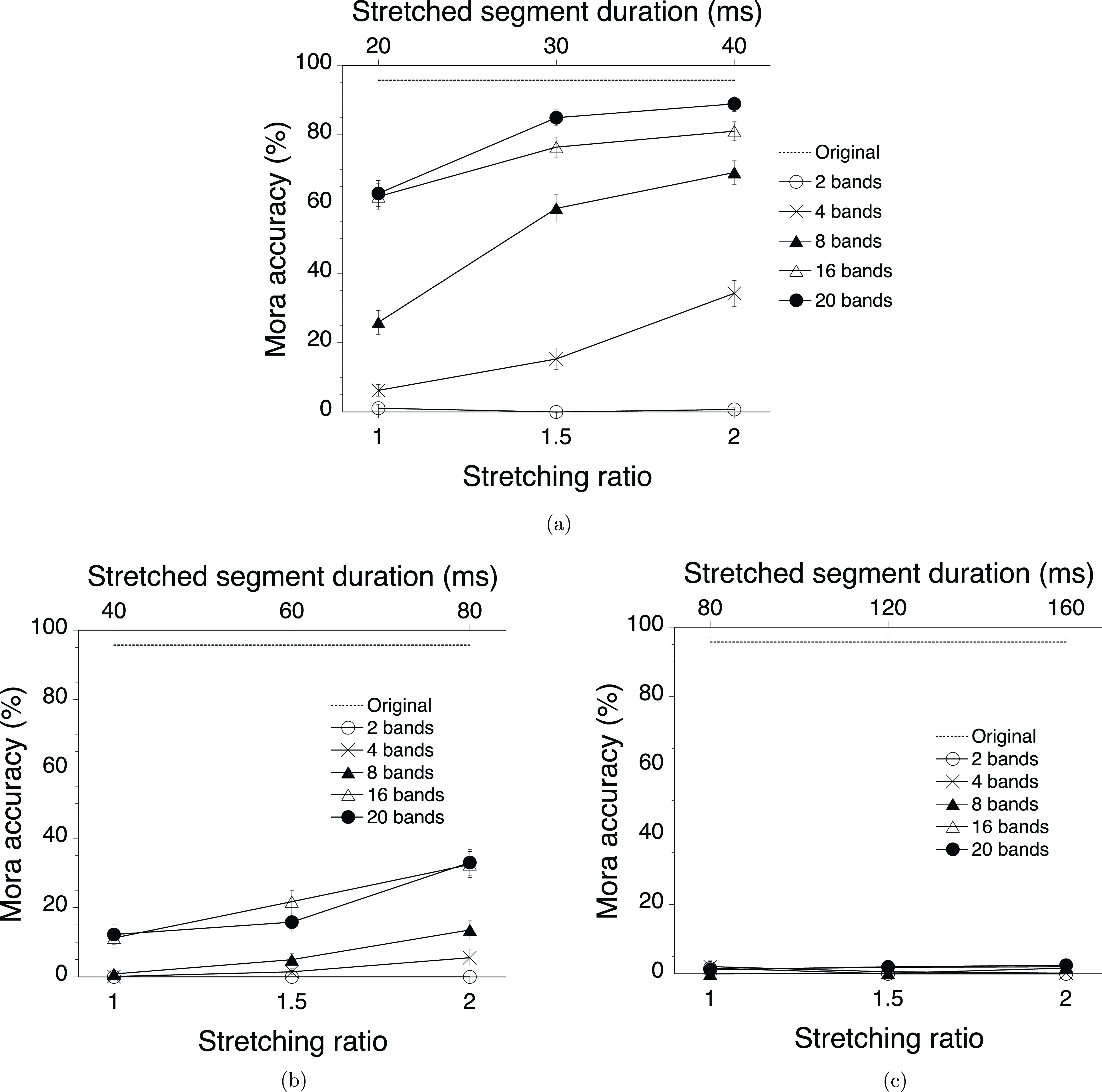
The mora accuracy data in Fig. 2 are replotted with the horizontal axes representing the stretching ratios. Panels represent the data for the original segment duration, (a) 20 ms, (b) 40 ms, and (c) 80 ms. The mora accuracy data for the original speech stimuli are presented in all panels as a reference. The error bars show the SEM.

Figures 2 and 3 show the experimental results, as well as represent the same data with different variables on the lower horizontal axes: original segment duration for Fig. 2 and stretching ratio for Fig. 3. The control condition with the original speech stimuli resulted in almost perfect performance (96%). The obvious trend in Fig. 2 for experimental conditions was that intelligibility (measured as the percentages of mora accuracy) decreased with increasing original segment duration and decreasing number of frequency bands except for the two-band condition, which always resulted in the floor performance. The effect of experimental manipulation looked large enough compared to the variability of the data shown in Figs. 2 and 3, despite the small number of repetitions (five sentences per condition in each participant).

The effect of stretching is most obvious in Fig. 3(a), which represents the data for the 20-ms original segment duration. For example, for the 20-band stimuli, stretching increased mora accuracy from 63% with the stretching ratio of 1.0 (no stretching) through 85% with the stretching ratio of 1.5 to 89% with the stretching ratio of 2.0, which was very close to the performance in the control condition. Although the performance generally went down as the number of frequency bands decreased, the improvements in performance with stretching were obvious except for the two-band stimuli. The same tendency was still observable to a smaller extent for the 40-ms original segment duration [Fig. 3(b)], whereas all performances went down to the floor for the 80-ms original segment duration [Fig. 3(c)].

To sum up, all three variables manipulated in the experiment, i.e., original segment duration, number of frequency band, and stretching ratio, were effective in altering intelligibility except for the two-band stimuli.

These observations were supported by the analysis using a generalized linear mixed model (GLMM) with a logistic linking function as implemented in an add-in for jmp Pro (SAS Institute Inc., 2021). In the following analysis, the data for the two-band stimuli were excluded because it was impossible to perform a proper statistical analysis with the data, which consisted of zeros in almost all of the conditions. Thus, the data for the 4-, 8-, 16-, and 20-band stimuli were analyzed for fixed effects of number of frequency band, original segment duration, stretching ratio (all categorical predictors) and their interactions, and random effects of listener and sentence. All of the statistical effects had a *p* level smaller than 0.001 unless reported otherwise. This model revealed the following effects with a corrected Akaike's information criterion (AICc) of 3003.7: number of frequency band [ F(3,1690)=74.32], original segment duration [ F(2,1881)=6.13, *p *=* *0.002], stretching ratio [ F(2,1903)=37.09], number of frequency band × original segment duration [ F(6,1895)=8.51], number of frequency band × stretching ratio [ F(6,1942)=4.58], original segment duration × stretching ratio [ F(4,1879)=4.19, *p *=* *0.002], and number of frequency band × original segment duration ×stretching ratio [ F(12,1891)=1.89, *p *=* *0.031].

## IV. DISCUSSION

### A. Replication of the preliminary results

The present results successfully replicated the results in the preliminary study by Ueda *et al.* (2021, experiment 1). The intelligibility of the interrupted mosaic speech stimuli (the stretching ratio of 1.0) with two and four bands was quite close to zero, irrespective of the original segment duration. The intelligibility for the 20-band stimuli was moderate (63%) at the 20-ms segment duration but was only 1% at the 80-ms segment duration as in the preliminary results.

### B. Intelligibility difference between uninterrupted and interrupted mosaic speech stimuli

In mosaic speech stimuli, the effect of interruption was quite devastating. The previous investigation by Nakajima *et al.* (2018) indicated that the intelligibility for uninterrupted mosaic speech stimuli with 20 bands stayed above 95% at the segment duration of 20 and 40 ms, and it declined to about 45% at the 80-ms segment duration. By contrast, the present investigation showed that the interruption reduced the intelligibility to 63%, 12%, and 1% for the original segment durations of 20, 40, and 80 ms, respectively, with 20 frequency bands. Considering, also, the mean deletion detection threshold (defined as mean median adjustments for clear deletion detection), which was measured for normal discourse, was 52 ms (Bashford and Warren, 1987), the intelligibility of mosaic speech stimuli is extremely vulnerable to interruption compared with the conventional speech stimuli used in the previous studies (e.g., Cherry and Wiley, 1967; Holloway, 1970; Kidd and Humes, 2012; Miller and Licklider, 1950; Powers and Wilcox, 1977; Shafiro *et al.*, 2018; Shafiro *et al.*, 2016; Ueda and Ciocca, 2021; Wiley, 1968).

### C. Insufficient speech fragments, failed auditory grouping, or reduced temporal resolution

The results clearly reject the reduced temporal resolution hypothesis because stretching the mosaic segments never reduced intelligibility. At the same time, the results highlight the importance of the frequency resolution and original segment duration: the number of frequency bands should be four or more, and the original segment duration should be equal to or shorter than 40 ms for the effective improvement of intelligibility by stretching the segments of interrupted mosaic speech.

Within these limits, speech intelligibility could reach 89% by just stretching the interrupted mosaic segments to fill in the gaps between the segments. This is a persuasive piece of evidence to support the failed auditory grouping hypothesis and reject the insufficient speech fragments hypothesis because stretching the mosaic segments should facilitate auditory grouping of the segments but should not increase the number of any of the speech fragments. Therefore, the results strongly suggest that in the interrupted mosaic speech stimuli, a poor grouping cue probably led to the deteriorated intelligibility.

The periodicity and temporal fine structure in normal speech mainly provide a grouping cue (Apoux and Healy, 2013; Apoux *et al.*, 2013) to combine the interrupted segments perceptually (Clarke *et al.*, 2016). In addition, spectral coherence and regularity in interruption also should be considered. Based on a series of systematic experiments in which checkerboard noise (Howard-Jones and Rosen, 1993) was used, Fogerty *et al.* (2018) and Fogerty *et al.* (2020) argued that periodic glimpsing (Buss *et al.*, 2004; Hall *et al.*, 2008; Ozmeral *et al.*, 2012) led to better performance than random spectrotemporal glimpsing and spectral coherence in maskers tended to result in better performance compared with temporal coherence. Furthermore, the periodic interruption for the vocoded speech stimuli can be seen as an interfering modulation which is superimposed over an envelope modulation in each frequency band conveying speech information. The situation can be regarded as modulation masking taking place (Houtgast and Steeneken, 1973; Takahashi and Bacon, 1992), and stretching the mosaic segments may reduce the prominence of these interfering envelope components, reducing modulation masking. One may conjecture that this reduction in modulation masking accounts for the improvement in the intelligibility for the stretched mosaic speech stimuli instead of auditory grouping. On the other hand, within the scope of the current investigation, the situation of reducing modulation masking always promotes auditory grouping as well. Considering that wider experimental results can be explained by auditory grouping than the reduction of modulation masking, we are inclined to presume that auditory grouping would be the key element. The arguments above can be generalized into understanding why the interruption of mosaic speech is so devastating for intelligibility and why the intelligibility for normal speech is so robust against periodic interruption.

Interestingly, it has been shown that speech understanding does not depend critically on the across-frequency temporal synchrony of amplitude modulation imposed on the speech material, based on the experiments in which masked thresholds were measured for nine narrow bands of Gaussian noise and speech (Buss *et al.*, 2003). Furthermore, the preliminary experiment (experiment 1; *n *=* *2) by Ueda *et al.* (2021) included the condition in which checkerboard mosaic speech stimuli were presented. The checkerboard mosaic speech stimuli were based on checkerboard speech stimuli, which were named after checkerboard noise (Howard-Jones and Rosen, 1993). The checkerboard speech stimuli were also produced with segmentation and interruption; however, the segmentation was performed in frequency by time units, and the interruption phase (on vs off) was switched across adjacent frequency bands. Thus, the checkerboard speech stimuli consisted of half of the original units and the other units were discarded. In that sense, the checkerboard speech stimuli and interrupted speech stimuli consisted of half of the original units, however, the intelligibility of the checkerboard speech stimuli showed a sharp contrast to the intelligibility of the interrupted speech stimuli. The intelligibility of the interrupted speech stimuli varied according to the segment duration, starting from nearly 100% at the shortest segment duration, i.e., 20 ms, and gradually declined to 55% at 320 ms. By contrast, 20-band checkerboard speech stimuli kept nearly 100% intelligibility irrespective of the segment duration, but two- or four-band checkerboard speech stimuli consistently showed lower intelligibility than the interrupted speech stimuli and reached the lowest intelligibility of 35% at the 160-ms segment duration (experiment 2; *n *=* *20). On the other hand, when the checkerboard speech stimuli were mosaicked, the intelligibility varied in a much the same way as in the interrupted mosaic speech stimuli: in most of the cases, the intelligibility was close to zero except for the stimuli with the 20-band and 20-ms segment duration, which brought 90% intelligibility (experiment 1). These results also support the failed auditory grouping hypothesis for the interrupted or checkered mosaic speech stimuli and, hence, highlight the importance of a grouping cue.

### D. Auditory grouping vs phonemic restoration by strong noise with masking potential

Our findings should be clearly distinguished from the previous findings, which were related to phonemic restoration by strong noise with masking potential (Warren, 1970, 2008; Warren and Warren, 1970). It is to be noted that the improvement of intelligibility observed in the current results was brought on by just stretching the mosaicked segments without introducing any external noise, including the condition in which 10-ms silent gaps were still present; whereas in the typical situation of phonemic restoration, the silent gaps were completely replaced with noise, and the noise should be clearly stronger than the interrupted speech to yield any improvement in intelligibility, hence, masking potential was the key (e.g., Cherry and Wiley, 1967; Holloway, 1970; Powers and Wilcox, 1977; Sasaki, 1980; Ueda and Ciocca, 2021; Wiley, 1968). Thus, in the current results, facilitating auditory grouping was the key not masking potential. Therefore, a reasonable interpretation of the current results would be that previously unavailable speech cues became available when the mosaicked segments were stretched and auditory grouping was promoted.

### E. The general limits necessary for speech perception: Four frequency bands and 40 ms

The results suggested that even when periodicity and temporal fine structure were removed by mosaicking, the intelligibility of interrupted mosaic speech stimuli could be improved by stretching the mosaicked segments and reducing the silent gaps, provided that the number of frequency bands was equal to or more than four and the original segment duration was equal to or less than 40 ms. These limits seem to have strong connections to the previous findings.

The limit of four frequency bands seems to be related to the number of frequency bands that is necessary for understanding noise-vocoded speech (Dorman *et al.*, 1997; Kishida *et al.*, 2016; Shannon *et al.*, 1995; Smith *et al.*, 2002; Ueda *et al.*, 2018; Ellermeier *et al.*, 2015). Because mosaic speech resembles noise-vocoded speech, it is logical that these two techniques of speech degradation share a common frequency resolution limit with regard to intelligibility. This limit is well accounted for by the factor analysis results (Ueda and Nakajima, 2017).

The limit of 40 ms appeared also as the limit for maintaining the ceiling performance of intelligibility for the speech stimuli in which slow (∼4 Hz) and rapid (∼33 Hz) modulations were desynchronized (Chait *et al.*, 2015). When speech rates of speakers were normalized, the same limit appeared as the maximum segment duration that locally time-reversed speech stimuli (Ueda *et al.*, 2017) or mosaic speech stimuli (Eguchi *et al.*, 2022) preserved intelligibility at the ceiling (cf. Nakajima *et al.*, 2018; Santi *et al.*, 2020; Ueda and Ciocca, 2021). The spectrotemporal limits of four frequency bands and 40 ms define a boundary of ∼4 cyc/kHz and 25 Hz in the modulation power spectrum (MPS). The previous studies reported that critical contribution to intelligibility came from the area of the low spectral (<2 cyc/kHz) and slow temporal (<10 Hz) modulation rates in the MPS (Elliott and Theunissen, 2009; Sohoglu and Davis, 2020; Venezia *et al.*, 2016). Ueda *et al.* (2021) also showed that the differences between original and checkerboard speech stimuli appeared in this area of the MPS, suggesting a strong connection between the intelligibility of the checkerboard speech stimuli and the low spectral and slow temporal modulation. By contrast, according to the MPS analysis on the stimuli used in the present study (see the supplementary material^1^), the effect of stretching the mosaicked segment duration appeared as demodulation in the area of rapid modulations (>20 Hz). These results are in accord with the *two-temporal-windows integration* (2TWI) model proposed by Poeppel and his colleagues (Chait *et al.*, 2015; Giraud and Poeppel, 2012; Giroud *et al.*, 2020; Poeppel, 2003; Sanders and Poeppel, 2007; Teng and Poeppel, 2020; Teng *et al.*, 2016), in which a short time-window of ∼20–30 ms and a long time-window of ∼200 ms work in parallel in the brain, reflecting the gamma rhythm and theta rhythm of neuronal oscillations in the auditory cortex. Further investigations are warranted to enlighten the connection between the model and perception of the interrupted mosaic speech stimuli.

Thus, the current findings together with the previous findings strongly suggest that the auditory system can process averaged spectral information within the window of 40 ms with equal to or more than four frequency bands for speech perception to attain high intelligibility. If the number of frequency bands falls short of the limit of the four frequency bands or the segment duration of original mosaic speech stimuli exceeds the 40-ms limit, the intelligibility improvement by stretching disappears.

## V. CONCLUDING REMARKS

Ever since the first systematic study by Miller and Licklider (1950), the use of interrupted speech stimuli to study speech perception has produced a vast amount of information regarding speech perception in adverse listening conditions and auditory continuity. The present investigation extends the use of interrupted speech to the study of auditory grouping. The extension was realized by introducing interrupted mosaic speech stimuli, which resulted in severely deteriorated intelligibility. Furthermore, the intelligibility was recovered with stretched mosaic segments within the limits of no less than four frequency bands and no longer than 40-ms original segment duration, although the corresponding original segments were unchanged and no improvement for temporal resolution was introduced. These results suggested that the devastating effect of interruption on intelligibility for mosaic speech stimuli stemmed from unsuccessful auditory grouping among separated segments with an abolished grouping cue. This also implies that the grouping cue should play an important role in perceiving interrupted normal speech stimuli as well.

## Footnotes

^1^
See the supplementary material at https://www.scitation.org/doi/suppl/10.1121/10.0013425 for the MPS analysis method and results.
